# Loss of the Coffin-Lowry syndrome-associated gene *RSK2* alters ERK activity, synaptic function and axonal transport in *Drosophila* motoneurons

**DOI:** 10.1242/dmm.021246

**Published:** 2015-11-01

**Authors:** Katherina Beck, Nadine Ehmann, Till F. M. Andlauer, Dmitrij Ljaschenko, Katrin Strecker, Matthias Fischer, Robert J. Kittel, Thomas Raabe

**Affiliations:** 1 Universityof Würzburg, Institute of Medical Radiation and Cell Research, Versbacherstraße 5, Würzburg D-97078, Germany; 2 University of Würzburg, Institute of Physiology, Department of Neurophysiology, Röntgenring 9, Würzburg D-97070, Germany; 3 University of Würzburg, Rudolf Virchow Center, DFG Research Center for Experimental Biomedicine, Josef-Schneider-Straße 2, Würzburg D-97080, Germany; 4 Freie Universität Berlin, Institute of Biology, Takusstraße 6, Berlin D-14195, Germany; 5 Max Planck Institute of Colloidals and Interfaces, Am Mühlenberg 1, Potsdam D-14476, Germany; 6 University Hospital Würzburg, Department of Psychiatry, Psychosomatics and Psychotherapy, Füchsleinstraße 15, Würzburg 97080, Germany

**Keywords:** *Drosophila*, Motoneuron, Neuromuscular junction, RSK, MAPK signaling, Synapse, Axonal transport

## Abstract

Plastic changes in synaptic properties are considered as fundamental for adaptive behaviors. Extracellular-signal-regulated kinase (ERK)-mediated signaling has been implicated in regulation of synaptic plasticity. Ribosomal S6 kinase 2 (RSK2) acts as a regulator and downstream effector of ERK. In the brain, RSK2 is predominantly expressed in regions required for learning and memory. Loss-of-function mutations in human *RSK2* cause Coffin-Lowry syndrome, which is characterized by severe mental retardation and low IQ scores in affected males. Knockout of *RSK2* in mice or the RSK ortholog in *Drosophila* results in a variety of learning and memory defects. However, overall brain structure in these animals is not affected, leaving open the question of the pathophysiological consequences. Using the fly neuromuscular system as a model for excitatory glutamatergic synapses, we show that removal of RSK function causes distinct defects in motoneurons and at the neuromuscular junction. Based on histochemical and electrophysiological analyses, we conclude that RSK is required for normal synaptic morphology and function. Furthermore, loss of RSK function interferes with ERK signaling at different levels. Elevated ERK activity was evident in the somata of motoneurons, whereas decreased ERK activity was observed in axons and the presynapse. In addition, we uncovered a novel function of RSK in anterograde axonal transport. Our results emphasize the importance of fine-tuning ERK activity in neuronal processes underlying higher brain functions. In this context, RSK acts as a modulator of ERK signaling.

## Abstract (precis)

**Summary:** Mutations in the human kinase RSK2 are associated with severe mental retardation (Coffin-Lowry syndrome). Here, the function of *Drosophila* RSK in synaptic plasticity is described, which might help in understanding the complex pathophysiology.

## INTRODUCTION

The p90 ribosomal S6 kinases (RSKs) are a family of serine-threonine kinases that act as downstream effectors of the RAS-mitogen-activated protein kinase (MAPK) pathway through direct interaction with the extracellular-signal-regulated kinase (ERK). In this way, RSK proteins link MAPK signaling to a multitude of substrate proteins. Thus, they regulate diverse cellular processes, such as gene expression, cell growth, proliferation and cell survival. The RSK family comprises four isoforms (RSK1-4) in vertebrates, which fulfil partly redundant but also isoform-specific functions (Romeo et al., 2012). In *Drosophila melanogaster* and in other invertebrates, only a single RSK isoform is expressed. The overall sequence conservation of *Drosophila* RSK to vertebrate RSK proteins shows no preference for a single isoform and is mainly restricted to the known functional domains.

Common structural features of all RSK proteins are two kinase domains (N-terminal kinase domain and C-terminal kinase domain), which are joined by a regulatory linker region, and a C-terminal docking site for ERK proteins. In response to stimulation of the MAPK pathway, ERK binds to RSK and thereby initiates a series of phosphorylation events. The C-terminal kinase domain becomes activated by ERK-mediated phosphorylation. Next, ERK and the C-terminal kinase domain phosphorylate several residues in the linker region of RSK. One of these sites is essential for binding 3′-phosphoinositide-dependent kinase-1, which, in turn, then phosphorylates and thereby activates the N-terminal kinase domain as the effector kinase. Finally, autophosphorylation of a serine residue near the ERK docking site by the N-terminal kinase domain promotes dissociation of ERK from RSK (Romeo et al., 2012). The conservation of all phosphorylation sites in all RSK proteins from different species suggests a common activation mechanism. However, recent studies in flies also suggested N-terminal kinase domain-independent functions of RSK (Kim et al., 2006; Tangredi et al., 2012). In addition to its function as a downstream effector of MAPK signaling, RSK acts as a localization determinant of ERK and can negatively feed back to prevent hyperactivation of the MAPK pathway (Romeo et al., 2012).

Deregulation of RSK function has been linked to several pathophysiological conditions in humans. Mutations in the human *RSK2* gene cause Coffin-Lowry syndrome (CLS), an X-linked disorder characterized by facial and progressive skeletal abnormalities and by severe intellectual disabilities in affected males. More than 140 mutations distributed over the *RSK2* gene have been identified in individuals with CLS; most of them are deletions or missense mutations that disrupt RSK function (Pereira et al., 2010). Despite the severity of the neurological defects, the processes regulated by RSK2 in the nervous system remain poorly defined.

**TRANSLATIONAL IMPACT**

**Clinical issue**

Coffin-Lowry syndrome (CLS) is a rare X-linked disorder, with an estimated incidence of 1:50,000 to 1:100,000. Affected males present with facial abnormalities and severe intellectual disabilities, with IQ scores ranging from 15 to 60. CLS is caused by inactivating mutations in the protein kinase RSK2, which acts as a regulator and mediator of the mitogen-activated protein kinase (MAPK) signaling pathway. This pathway has essential roles in cellular proliferation and differentiation, but the absence of major brain abnormalities in individuals with CLS suggests an additional involvement at the neurophysiological level. RSK2 is predominantly expressed in brain regions involved in learning and memory; however, the exact functions of RSK2 remain poorly understood. Behavioral defects are observed in RSK2 knockout mice and in *Drosophila* upon knockout of RSK, the single fly ortholog of vertebrate RSK proteins. In this study, the authors used the *Drosophila* neuromuscular system as a well-established model for excitatory glutamatergic synapses to study the physiological consequences of loss of RSK function.

**Results**

Consistent with previous findings, a general upregulation in activity of the final MAPK component, ERK, was observed in *RSK­*-deficient motoneurons in *Drosophila*. Intriguingly, the authors observed redistribution of activated ERK from synaptic terminals to the somata, suggesting that local signaling events are altered in the absence of RSK. To determine whether RSK is required for synaptic function, the authors performed immunohistochemical and electrophysiological experiments. They demonstrate that loss of RSK function impairs synaptic transmission and, interestingly, defects in anterograde transport of mitochondria were uncovered using *in vivo* imaging techniques.  Overall, their data indicate a postsynaptic requirement of RSK for efficient synaptic transmission, in line with data from studies in mice, but also uncover a presynaptic role. 

**Implications and future directions**

This study uncovers a multifaceted requirement of RSK2 for regulation of synaptic function and MAPK-dependent processes in neurons. An emerging common picture from animal models of CLS is a postsynaptic function of RSK2. In addition, the present study is the first to implicate RSK in anterograde axonal transport processes, the distribution of activated ERK and synaptic organization in motoneurons, suggesting an additional role for RSK2 in the presynaptic neuron. Exactly how RSK2 regulates presynaptic processes at the molecular level remains to be discovered. A major challenge will be to distinguish the ambivalent functions of RSK2 both as a downstream mediator but also as a negative regulator of MAPK signaling in these processes, and to explore potential differential effects on local MAPK signaling. Only with this knowledge can our understanding of the pathophysiology of CLS be improved further.

Animal knockout models have been established for mouse *RSK2* and *Drosophila RSK*. In analysis of these mutants, diverse phenotypes were identified. Distinct deficits in different behavioral paradigms, such as spatial learning, long-term spatial memory and consolidation of fear memory in mice (Poirier et al., 2007; Morice et al., 2013) and in olfactory, operant and spatial learning in flies, were observed (Putz et al., 2004; Neuser et al., 2008). *Drosophila* RSK is also required in clock neurons to maintain normal circadian periodicity (Akten et al., 2009; Tangredi et al., 2012). No gross alterations in brain structure of *RSK2* mutants are evident, although a decrease in differentiation of cortical radial progenitors into neurons was observed in mice, which indicated a function of RSK2 in neurogenesis (Dugani et al., 2010). At the cellular level, survival of isolated spinal motoneurons from *RSK2*-deficient mice was not affected, but axonal outgrowth was increased (Fischer et al., 2009a). In the dentate gyrus, alterations in the morphology of dendritic spines were observed (Morice et al., 2013). Physiological changes include an increase of cortical dopamine levels, which is accompanied by elevated expression of the dopamine receptor DrD2L (Pereira et al., 2008). In the hippocampus, upregulated expression of the GluR2 subunit of AMPA-type glutamate receptors (GluRs) is correlated with changes in channel properties, synaptic transmission and long-term potentiation-induced gene expression (Mehmood et al., 2011; Morice et al., 2013). Increased GluR2 expression is caused by elevated ERK-mediated transcriptional activity, which results from a lack of RSK feedback inhibition of the MAPK pathway (Schneider et al., 2011; Mehmood et al., 2013). Increased ERK activity was also observed in *RSK2*-deficient motoneurons (Fischer et al., 2009a). In *Drosophila*, RSK negatively regulates ERK-dependent differentiation processes during eye development by acting as a cytoplasmic localization determinant of ERK (Kim et al., 2006). Altogether, these studies provide evidence for a multilayered, cell-type-specific function of RSK2 in the nervous system.

Owing to its relative simplicity and repetitive organization, the *Drosophila* larval neuromuscular system is a powerful model for the study of synapse formation, neurotransmission and synaptic plasticity at the structural, physiological and molecular levels (Menon et al., 2013). At each individual muscle, the neuromuscular junction (NMJ) shows a fairly reproducible branching pattern, with terminal structures called boutons, each of which harbors a number of presynaptic active zones as neurotransmitter release sites and opposed postsynaptic densities containing receptor clusters. Unlike the cholinergic NMJ in vertebrates, the *Drosophila* NMJ is glutamatergic and contains ionotropic GluRs that are homologous to non-NMDA-type GluRs in excitatory synapses of the vertebrate brain. Thus, defects in the fly neuromuscular system caused by loss of RSK function can provide insights into RSK2 functions at excitatory synapses of the vertebrate brain. Based on previous genetic experiments, which indicated a negative regulatory function of RSK in MAPK signaling (Fischer et al., 2009b), we now show that loss of RSK causes several distinct defects in motoneurons. First, we observed pronounced redistribution of activated ERK from synaptic sites to the somata. Second, we observed aberrant accumulations of synaptic components and defects in anterograde axonal transport of mitochondria. Third, changes in the number of active zones and postsynaptic receptor fields are evident, which are correlated with impairment of synaptic transmission. These findings provide new insights on RSK function in synaptic plasticity, which might help in understanding the complex pathophysiology of *RSK2* mutants in vertebrates and in human disease.

## RESULTS

### Presynaptic localization of RSK

**Fig. 1.**
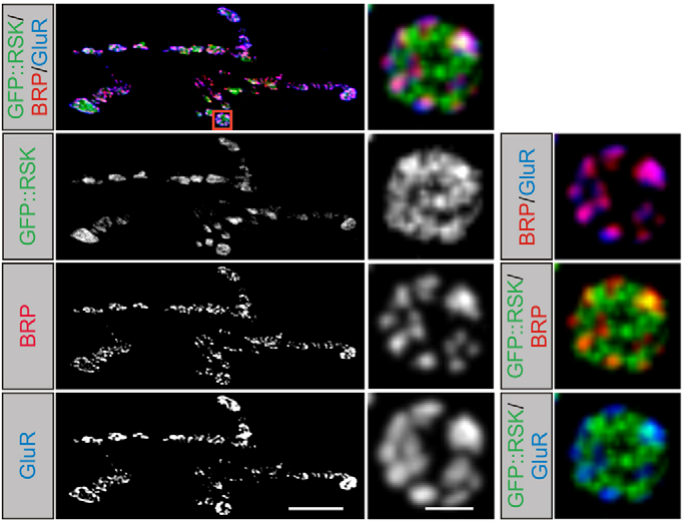
**Localization of RSK at the presynapse.** Left panels: projection view of a neuromuscular junction at muscle 6/7 stained for Bruchpilot (BRP; red), glutamate receptor II subunit D (GluRIID; blue) and transgenic GFP::RSK (green) expressed in motoneurons with the driver line *D42-Gal4*. Scale bar: 10 µm. Right panels: detail of a single bouton shown in the red box. A single confocal section is shown. BRP marks several active zones in the bouton, which are apposed by GluR fields marked by GluRIID. Presynaptic GFP::RSK staining is also seen outside active zones. Scale bar: 2 µm.

Based on previous genetic studies, which implicated a function of *Drosophila* RSK in motoneurons (Fischer et al., 2009b), we first determined the subcellular localization of RSK. Although available antisera against *Drosophila* RSK detected the endogenous protein on western blots (Putz et al., 2004), the antisera are not sensitive enough to detect endogenous RSK by immunohistochemistry of larval brain and body-wall preparations. Therefore, transgenic flies expressing green fluorescent protein (GFP)-tagged RSK under control of the *UAS*-enhancer (*UAS-RSK::GFP*) were established and crossed with the motoneuron driver line *D42-Gal4*. NMJs at muscle 6/7 in segment A2 of late third instar larval brain and body-wall preparations were examined with antibodies detecting presynaptic active zone and postsynaptic density components. Bruchpilot (BRP) is the fly ortholog of vertebrate CAST/ELKS and a major constituent of the cytomatrix associated with active zones (Wagh et al., 2006). Postsynaptic densities were marked with an antibody against the common D subunit of the heteromeric glutamate receptor II (GluRIID; Qin et al., 2005). GFP::RSK localizes presynaptically in boutons of the NMJ (Fig. 1). GFP immunoreactivity was not restricted to active zones outlined by BRP, but instead showed a broader distribution pattern in the presynaptic cytosol (Fig. 1). When GFP::RSK was expressed with the muscle-specific driver line *Gal4-DMef2*, a perinuclear and a faint ubiquitous staining was observed, without enrichment at postsynaptic sites (Fig. S1).

### Loss of RSK affects ERK activity and subcellular localization in motoneurons

**Fig. 2.**
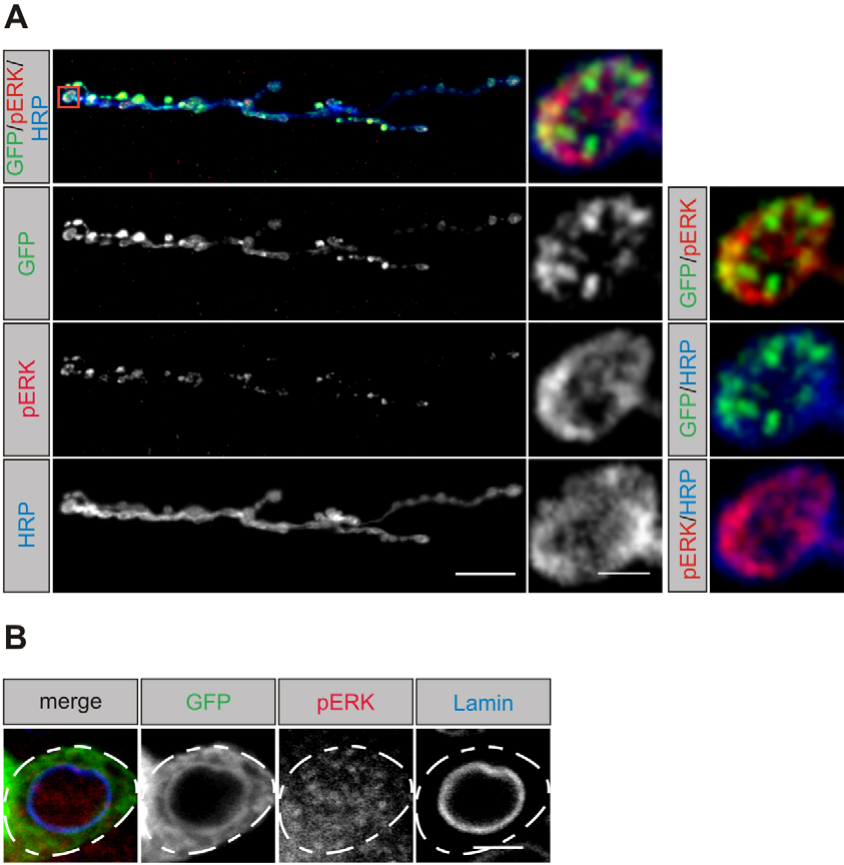
**Localization of RSK and pERK in motoneurons.** (A) Left panels: projection view of a neuromuscular junction at muscle 6/7 stained for GFP::RSK expressed with *D42-Gal4* (green), phosphorylated ERK (pERK; red) and horseradish peroxidase (HRP; blue). Scale bar: 20 µm. Right panels: single confocal section of a bouton shown in the red box. GFP::RSK and pERK partly colocalize at presynaptic sites. Scale bar: 2 µm. (B) Staining of the soma of a motoneuron (outlined by dashed line) for GFP::RSK (green), pERK (red) and Lamin (blue) as a nuclear membrane marker. RSK and pERK localize to the perikaryon. In addition, pERK staining is also seen in the nucleus. Scale bar: 2 µm.

Previous studies identified *Drosophila* RSK as a direct interaction partner of ERK (Kim et al., 2006). Binding of ERK is essential for RSK function in eye and wing development and for circadian behavior, and it stimulates activation of the C-terminal kinase domain by phosphorylation. In contrast to biochemical studies with vertebrate RSK proteins, subsequent activation of the N-terminal kinase domain as the effector kinase for phosphorylation of substrate proteins is dispensable, at least for these processes (Kim et al., 2006; Tangredi et al., 2012). Based on genetic studies using different combinations of loss- and gain-of-function mutations, it was concluded that in the case of the developing *Drosophila* eye, RSK acts as a cytoplasmic anchor for ERK to regulate nuclear entry and thereby ERK-dependent transcription (Kim et al., 2006). By contrast, biochemical studies in vertebrates implicate a function of RSK2 in feedback inhibition of the MAPK pathway (Romeo et al., 2012). Corresponding biochemical evidence for *Drosophila* RSK is still missing. Given that genetic interaction experiments also indicated a negative regulatory function of RSK in the neuromuscular system (Fischer et al., 2009b), we investigated whether RSK and ERK colocalize in motoneurons and whether loss of RSK has an impact on ERK activity or its subcellular localization. Immunostaining of *Drosophila* larval motoneurons using a mouse monoclonal antibody against double-phosphorylated (activated) ERK (pERK) gave inconsistent results, both in our hands (data not shown) and in other studies (Koh et al., 2002; Wairkar et al., 2009). We therefore first re-evaluated localization of pERK by using a rabbit monoclonal antibody (#4370; Cell Signaling) in combination with staining for GFP::RSK expressed in motoneurons by *D42-Gal4*. At the NMJ, pERK and RSK accumulate at presynaptic sites in a spot-like pattern (Fig. 2A). Colocalization is evident for many but not for all puncta, indicating an interaction of the proteins at selective sites of the presynapse. However, localization of both proteins is not restricted to the presynapse. GFP::RSK was also detected in the perikaryon, but not in the nucleus of motoneurons, as distinguished by staining for the nuclear membrane marker Lamin, whereas weak pERK staining was observed in both the perikaryon and the nucleus (Fig. 2B). Predominant cytoplasmic localization of RSK was also previously shown for ectopically expressed RSK in clock neurons of the fly circadian circuit (Akten et al., 2009).

**Fig. 3.**
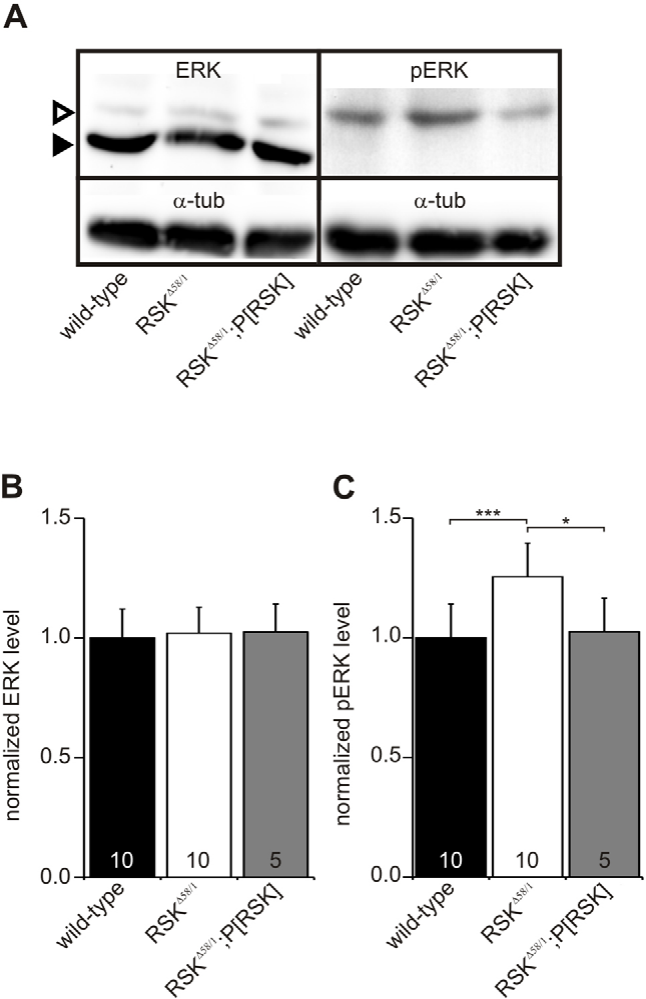
**Loss of RSK increases ERK activity.** (A) Western blots of lysates from third larval instar ventral ganglia probed with antibodies against total ERK, phosphorylated ERK (pERK) and α-tubulin (α-tub). The ERK antibody detects non-phopshorylated (arrowhead) and phosphorylated ERK (open arrowhead). (B,C) Quantification of ERK (B) and pERK (C) levels normalized to α-tubulin from at least five independent biological experiments (denoted in the bars). Compared with wild type, the level of pERK but not of ERK is increased in *RSK^Δ58/1^* and returned to wild-type levels in the presence of the *P[RSK]* transgene. *P≤0.05 and ***P≤0.001.

Using a previously characterized null mutant for *RSK* (*RSK^Δ58/1^*; Putz et al., 2004), we next determined whether complete loss of RSK alters overall levels of pERK. To enrich for the population of motoneurons, we restricted the analysis to the ventral ganglion. Ventral ganglia were dissected from third instar larval central nervous system preparations, and lysates were analyzed by western blot using antibodies against ERK and pERK, with α-tubulin for normalization (Fig. 3A). Normalized ERK levels showed no difference between wild-type and *RSK^Δ58/1^* preparations (Fig. 3B; wild type=1.00±0.12; *RSK^Δ58/1^*=1.02±0.11), indicating that loss of RSK has no influence on overall ERK levels. By contrast, pERK levels were significantly increased in *RSK^Δ58/1^* compared with wild type (Fig. 3C; wild type=1.00±0.14; *RSK^Δ58/1^*=1.25±0.16, *P*<0.001)*.* To validate the observed changes in pERK activity, a 20 kb genomic rescue construct encompassing the 6 kb *RSK* transcription unit (in the following, termed *P[RSK]*) was introduced into a *RSK^Δ58/1^* background. In *RSK^Δ58/1^*;*P[RSK]* animals, normalized pERK levels became significantly different from *RSK^Δ58/1^* (Fig. 3A,C; 1.02±0.15, *P*<0.05 versus *RSK^Δ58/1^*) and returned to wild-type levels. In summary, the results are in line with previous genetic analyses, which indicated a function of RSK as a negative regulator of the ERK/MAPK signaling pathway (Kim et al., 2006).

**Fig. 4.**
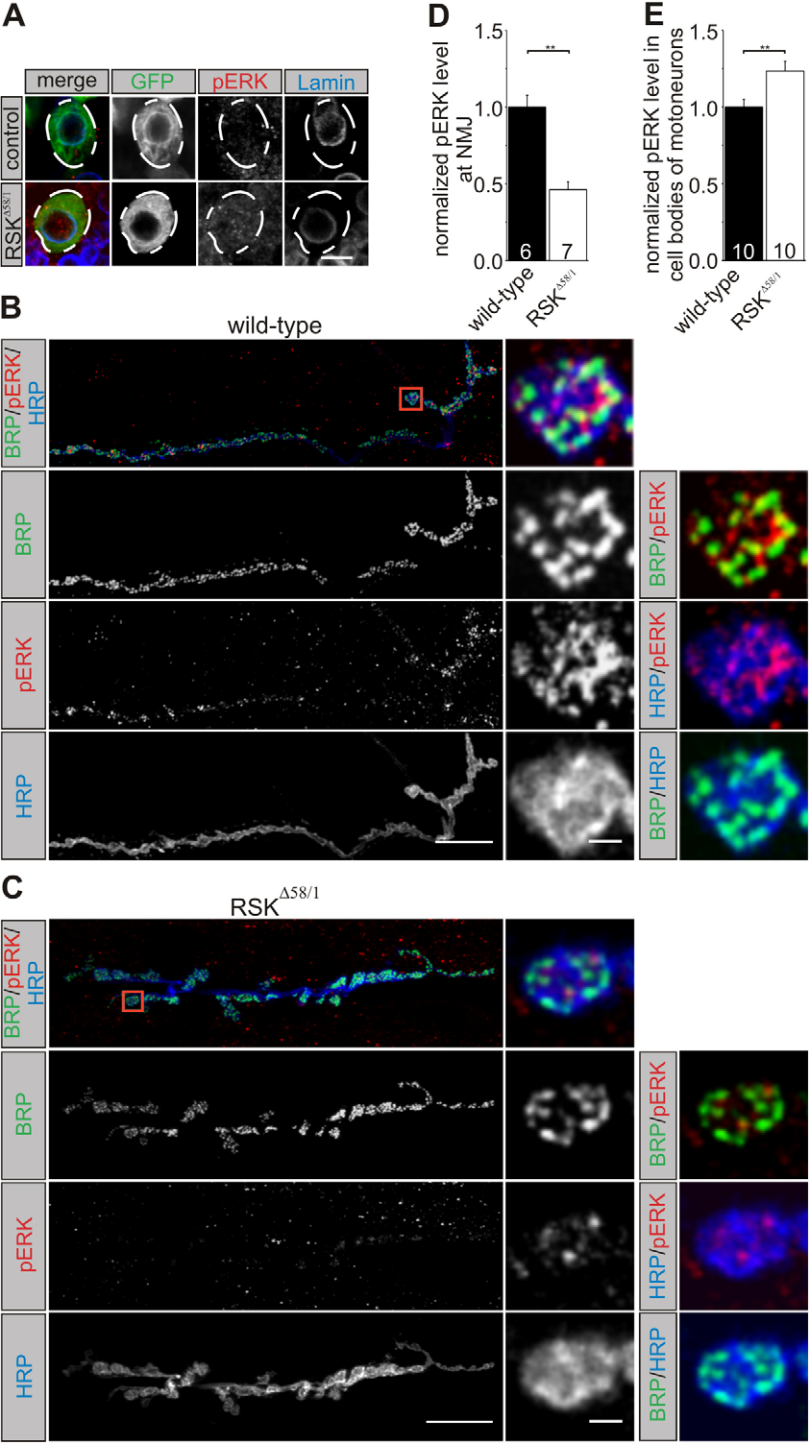
**Loss of RSK affects pERK levels and subcellular distribution in motoneurons.** (A) Somata of motoneurons in controls or *RSK^Δ58/1^* animals were identified by *D42-Gal4*-driven expression of the *mCD8::GFP* marker. Motoneuron somata are outlined by dashed lines. Staining for GFP (green), Lamin (blue) and phosphorylated ERK (pERK; red) showed increased pERK signals in the perikaryon and nucleus of *RSK^Δ58/1^* motoneurons. Scale bar: 2 µm. (B,C) Left panels: projection view of neuromuscular junctions (NMJs) from wild type (B) and *RSK^Δ58/1^* (C) stained for Bruchpilot (BRP; green), pERK (red) and horseradish peroxidase (HRP; blue), which labels the complete neuronal terminal. Scale bar: 20 µm. Right panels: single confocal sections of boutons (red boxes) are shown in detail. Compared with wild type (B), staining of pERK is nearly depleted at the NMJ of *RSK^Δ58/1^* animals (C). Scale bar: 2 µm. (D,E) pERK signal intensities were quantified at the NMJ (D) and in cell bodies (E) from whole central nervous system and body-wall preparations from control and *RSK^Δ58/1^* animals and normalized to mCD8::GFP expressed in motoneurons (see Fig. S2). The number of preparations analyzed is denoted in the bars. **P≤0.01.

Does loss of RSK have an impact on subcellular pERK localization in motoneurons? In order to identify motoneuron cell bodies in the ventral ganglia reliably, a membrane-tethered variant of GFP (mCD8::GFP) was expressed in motoneurons either in a wild-type or an *RSK^Δ58/1^* background. Consistent with the result shown in Fig. 2B, we again observed weak nuclear and perinuclear pERK staining for wild-type motoneurons (Fig. 4A). By contrast, pERK signal intensities in the nucleus and the perikaryon are elevated in *RSK^Δ58/1^* mutants (Fig. 4A). Although pERK levels appeared to be increased, no obvious change in the nuclear versus cytoplasmic distribution pattern was evident. This result is different from findings in differentiating cells of the developing eye, where loss of RSK affects localization of pERK but not overall pERK protein levels (Kim et al., 2006). Strikingly, elevated pERK levels were observed only in the somata of *RSK^Δ58/1^* motoneurons. At the NMJ, pERK staining was clearly visible in wild-type motoneurons (Fig. 4B), whereas a strong reduction of pERK staining was evident in the mutant (Fig. 4C). For a quantitative comparison of pERK levels, the experiment was repeated with complete central nervous system and body-wall preparations from control (*n*=10) and *RSK^Δ58/1^* animals (*n*=10) expressing mCD8::GFP in motoneurons. Analysis was done at the level of cell bodies and NMJs in identical conditions. For each preparation, pERK and GFP intensity levels were determined in 10 individual cell bodies and from the NMJ at muscle 6/7 in abdominal segment A2. Compared with control animals, normalized pERK values in *RSK^Δ58/1^* were strongly decreased at the NMJ and significantly increased in cell bodies (Fig. 4D,E; Fig. S2). From this analysis, we conclude that RSK is not only required for regulation of pERK levels, but also determines the distribution of pERK within different motoneuron compartments. This implies that deregulation of motoneuron function in *RSK^Δ58/1^* animals could be caused by altered ERK activity within distinct cellular compartments. One possibility would be enhanced ERK activity in cell bodies, leading to transcriptional activation of target genes. Alternatively, mutants might suffer from a lack of ERK activity at presynaptic sites. Given that ERK has been implicated in regulation of synaptic properties at the *Drosophila* NMJ (Koh et al., 2002; Wairkar et al., 2009), we focused the further phenotypic and physiological analysis of *RSK^Δ58/1^* on the NMJ.

### Loss of RSK affects NMJ size, synapse numbers and function

**Fig. 5.**
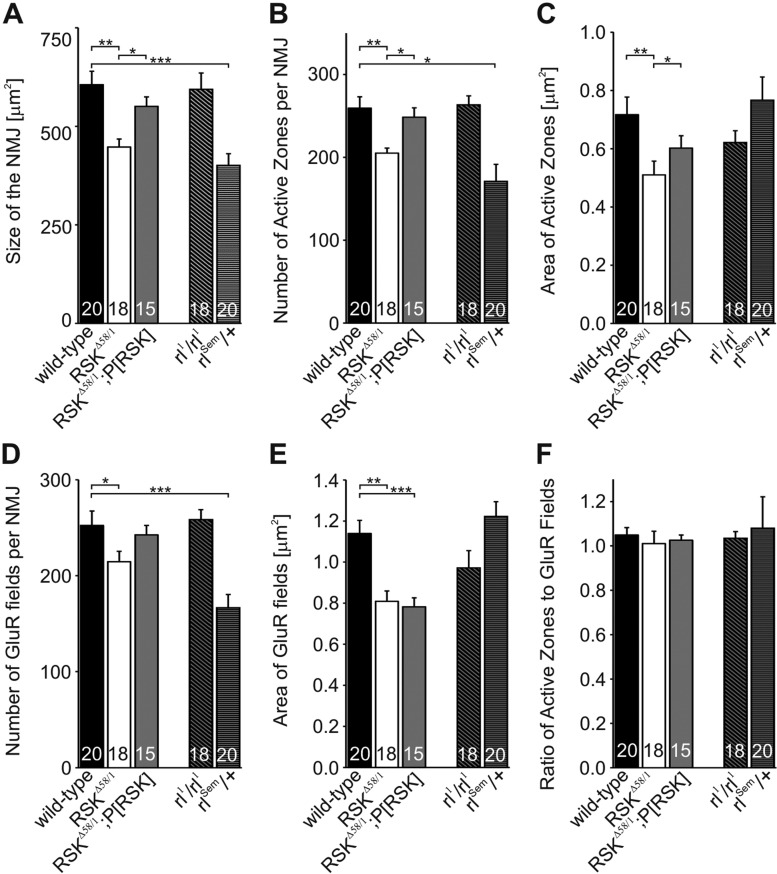
**Effects of *RSK^Δ58/1^* on neuromuscular junction size, active zones and receptor fields.** Quantifications were done at the neuromuscular junctions (NMJs) of muscle 6 and 7 in abdominal segment A2 or A3. (A) NMJ size was determined by outlining horseradish peroxidase (HRP) staining. (B,C) Bruchpilot (BRP) was used to quantify the number (B) and area (C) of active zones per NMJ. (D,E) Glutamate receptor II subunit D (GluRIID) was used to determine the number (D) and area (E) of glutamate receptor (GluR) fields per NMJ. Compared with wild type, all measured parameters are significantly reduced in *RSK^Δ58/1^*. (F) The ratio of BRP to GluRIID is depicted and does not differ between genotypes. NMJ size and presynaptic defects of *RSK^Δ58/1^* are rescued by *P[RSK]*, whereas postsynaptic defects are not rescued or partly rescued. (A-F) Animals homozygous for the hypomorphic ERK allele *rl^1^* exhibit wild-type NMJ morphology. By contrast, the activated variant *rl^Sem^* caused a significant decrease in NMJ size (A), number of active zones (B) and GluR fields (D). The number of individual larvae per genotype analyzed is denoted in the bars. *P≤0.05, **P≤0.01 and ***P≤0.001.

**Fig. 6.**
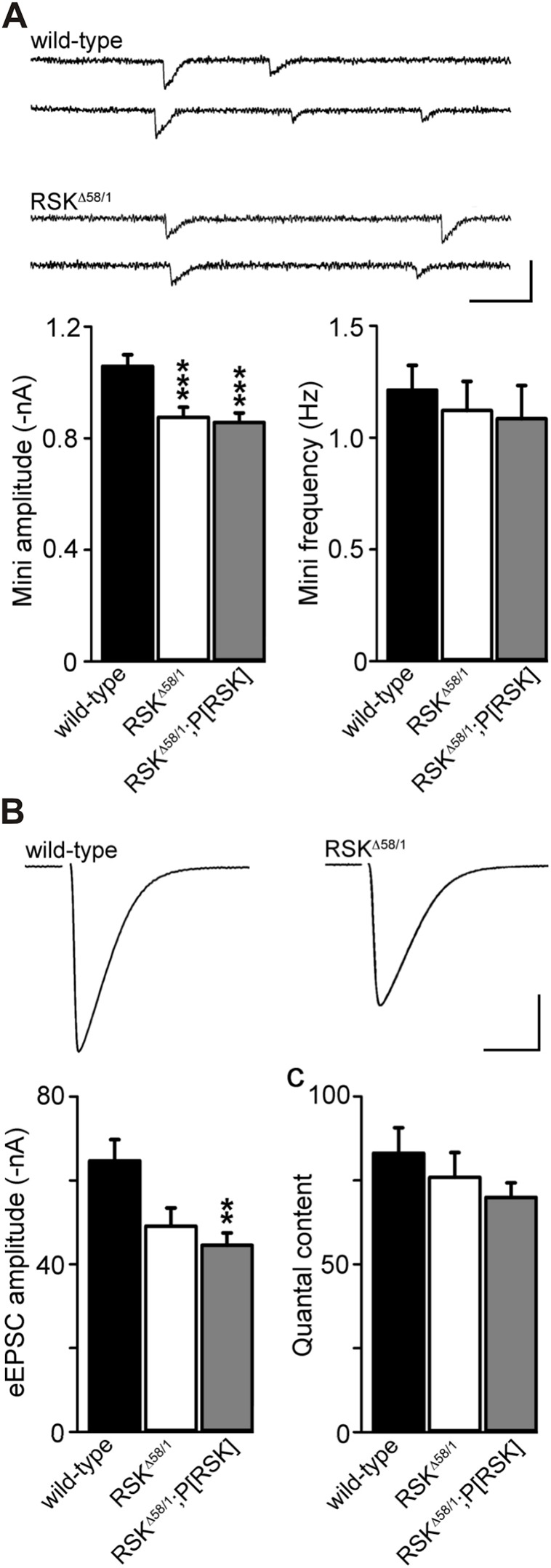
**Electrophysiological characterization of *RSK* mutant synapses.** (A) Example traces and quantification of miniature excitatory junctional currents (minis) recorded in two-electrode voltage-clamp recordings from the larval neuromuscular junction. The average mini amplitude was significantly smaller in *RSK^Δ58/1^* and could not be restored by employing a genomic rescue construct (*RSK^Δ58/1^;P[RSK]*). Mini frequency was not affected by loss of RSK. (B) Representative evoked excitatory postsynaptic currents (eEPSCs; stimulation artifact removed for clarity) during low-frequency nerve stimulation (0.2 Hz) and quantification of amplitudes. (C) Quantal content was comparable in all genotypes. Scale bars: (A) 2 nA (nanoampere), 50 ms; (B) 20 nA, 10 ms. **P≤0.01 and ***P≤0.001.

Loss of UNC-51, a negative regulator of ERK, leads to a decrease in the number and density of synapses, apposition defects of GluR fields with presynaptic active zones and impairment of evoked transmitter release (Wairkar et al., 2009). Thus, regulation of ERK activity provides one potential mechanism for synapse-specific control of protein composition and transmission. Taking advantage of the stereotypic branching pattern and terminal-specific number of boutons at muscle 6/7 in abdominal segments A2 and A3, we performed staining for BRP, GluRIID and the presynaptic membrane marker horseradish peroxidase (HRP). We quantified the following parameters in wild-type and *RSK^Δ58/1^* animals: overall size of the NMJs, the number of active zones and the number of GluR fields, and the average areas of single active zones and GluR fields (Fig. 5). For the total NMJ size (Fig. 5A), a significant decrease was observed in *RSK^Δ58/1^* animals compared with the wild-type control (NMJ size wild type=605.5±34.1 µm^2^; *RSK^Δ58/1^*=447.6±20.2 µm^2^, *P*<0.01). At the presynaptic level, the number (Fig. 5B) and area (Fig. 5C) of active zones per NMJ were significantly reduced in *RSK^Δ58/1^* (active zone number wild type=259.4±13.7 and *RSK^Δ58/1^*=205.0±6.1, *P*<0.01; active zone area wild type=0.72±0.06 µm^2^ and *RSK^Δ58/1^*=0.48±0.04 µm^2^, *P*<0.01). The presynaptic defects correlate with a reduction in the number (Fig. 5D) and area (Fig. 5E) of GluR fields (number wild type=257.0±15.0 and *RSK^Δ58/1^*=214.6±10.8, *P*<0.05; area wild type=1.14±0.06 µm^2^ and *RSK^Δ58/1^*=0.81±0.05 µm^2^, *P*<0.01). Calculation of the ratio of active zones to GluR fields (Fig. 5F) showed no difference between wild type and *RSK^Δ58/1^*, indicating that the general assembly and integrity of synaptic connections is not disturbed in the mutant. Indeed, no apposition defects of presynaptic BRP and postsynaptic GluRIID were observed, which distinguishes the *RSK^Δ58/1^* from the *UNC-51* loss-of-function phenotype. In the *UNC-51* mutant, postsynaptic receptor fields are often unapposed to active zones (Wairkar et al., 2009). To validate the pre- and postsynaptic phenotypes seen in *RSK^Δ58/1^*, the independent *RSK* deletion mutant, *RSK^D1^* (Kim et al., 2006), was analyzed. Indeed, the same phenotypes were observed (Fig. S3). In addition, we combined the *RSK^Δ58/1^* mutant with the genomic *P[RSK]* transgene for rescue experiments. Within these animals, values for NMJ size (Fig. 5A: 550.6±24.1 µm^2^, *P*<0.05 versus *RSK^Δ58/1^*), number (Fig. 5B: 248.4±11.3, *P*<0.05 versus *RSK^Δ58/1^*) and the area of active zones (Fig. 5C: 0.60±0.04 µm^2^, *P*<0.05 versus *RSK^Δ58/1^*) were significantly different from *RSK^Δ58/1^*, but not from wild-type measurements. Thus, the presynaptic phenotypes could be fully rescued with this construct. On the postsynaptic site, however, the number of GluR fields (Fig. 5D: 242.5±9.9) was not significantly different from wild type or from *RSK^Δ58/1^*. Measurements for the area of GluR fields still corresponded to *RSK^Δ58/1^* mutant values (Fig. 5E: 0.78±0.04 µm^2^). One potential explanation for rescue of presynaptic but not of postsynaptic phenotypes could be an incomplete expression pattern of transgenic RSK or expression of RSK at non-physiological levels. The lack of a suitable antibody for immunohistochemistry did not allow us to test the first possibility. However, RSK protein levels derived from this transgene did not exactly match endogenous RSK expression level (data not shown). Obviously, RSK levels must be tightly regulated to accomplish normal synapse function (see also Fig. 6).

Furthermore, we investigated how the observed decrease in NMJ size and overall number of active zones and GluR fields in both *RSK* mutants relate to the previously reported increase in the number of synaptic boutons (Fischer et al., 2009b). Consistent with this previous report, in the present study bouton numbers were significantly increased in *RSK^Δ58/1^*, and to the same degree in *RSK^D1^* (Fig. S4A). Notably, in both mutants, many boutons were much smaller in size (Figs S3A and S4B) and contained correspondingly fewer synaptic sites (Fig. S4C); therefore, the number of synapses per bouton area remained unchanged, as previously reported (Fischer et al., 2009b). In summary, the increase of bouton number at the NMJ of *RSK^Δ58/1^* and *RSK^D1^* animals is counteracted by a strong decrease in bouton size and the number of synapses per bouton, resulting in an overall reduction of NMJ size and of total active zone and GluR numbers.

The involvement of RSK proteins in feedback inhibition of the MAPK pathway but also as only one of many downstream targets of ERK signaling (Romeo et al., 2012) impeded prediction of NMJ phenotypes by interfering with ERK activity. *Drosophila* ERK is encoded by *rolled* (*rl*). Complete loss-of-function mutations in *rl* cause lethality (Biggs et al., 1994). Impairment of ERK function by the homozygous viable hypomorphic *rl^1^* allele showed no significant effect on all measured NMJ parameters (Fig. 5A-F), as published previously (Wairkar et al., 2009). On the contrary, a dominant gain-of-function allele of *rl*, *rl^Sem^*, resulted in moderately elevated ERK activity levels (Brunner et al., 1994; Oellers and Hafen, 1996). Heterozygous *rl^Sem^*/+ and homozygous *RSK^Δ58/1^* animals might display similar NMJ phenotypes as a result of increased ERK activity. However, this only holds true for NMJ size (Fig. 5A: 400.8±29.8 µm^2^, *P*<0.001 versus wild type), number of active zones (Fig. 5B: 170.9±20.6, *P*<0.001 versus wild type) and GluR fields (Fig. 5D: 166.5±13.8, *P*<0.001 versus wild type), whereas active zone and GluR areas in *rl^Sem^*/+ were not significantly different from wild-type values (Fig. 5C,E). Given that RSK is unable to bind the Rl^Sem^ protein (Kim et al., 2006), several interpretations are possible; phenotypes arise because of failure to transmit the signal via RSK or enhanced activation of other substrate proteins by Rl^Sem^. However, the presence of a wild-type copy of *rl* in *rl^Sem^*/+ animals raises the additional possibility that some phenotypes are masked because endogenous RSK function is still under control of ERK. Resolving these issues by analysis of *RSK^Δ58/^;rl^1^/rl^1^* and *RSK^Δ58/1^;rl^Sem^*/+ double mutants (data not shown) has provided no coherent picture so far.

To analyze whether the observed morphological changes correlate with altered synaptic transmission, we performed two-electrode voltage-clamp recordings at larval NMJs (Fig. 6). The amplitude of miniature excitatory junctional currents (minis), the postsynaptic response to spontaneous fusions of single glutamate-filled synaptic vesicles, was significantly reduced in *RSK^Δ58/1^* mutants (Fig. 6A; wild type=−1.06±0.04 nA, *n*=15; *RSK^Δ58/1^*=−0.88±0.04 nA, *n*=14, rank sum test *P*≤0.001 versus wild type) and remained so upon RSK re-expression (Fig. 6A; *RSK^Δ58/1^;P[RSK]*=−0.86±0.03 nA, *n*=15, rank sum test *P*≤0.001 versus wild type). These data are consistent with the reduced area of glutamate receptor fields, which could not be rescued and indicates diminished postsynaptic sensitivity of *RSK* mutants. By contrast, we observed no functional change in presynaptic properties. The frequency of minis was similar to wild-type animals (Fig. 6A; wild type: 1.21±0.11 Hz, *n*=15; *RSK^Δ58/1^*: 1.12±0.13 Hz, *n*=14, rank sum test *P*=0.83 versus wild type; *RSK^Δ58/1^;P[RSK]*=1.09±0.15 Hz, *n*=15, rank sum test *P*=0.11 versus wild type). The reduction in the amplitude of evoked excitatory postsynaptic currents (eEPSCs; Fig. 6B; wild type=−64.68±5.07 nA, *n*=15; *RSK^Δ58/1^*=−49.09±4.35 nA, *n*=14, rank sum test *P*=0.064 versus wild type; *RSK^Δ58/1^;P[RSK]*=−44.54±2.93 nA, *n*=15, rank sum test *P*=0.003) could be ascribed to the smaller minis. Correspondingly, the number of vesicles released per action potential (quantal content) was comparable in all three genotypes (Fig. 6C; 83±8, *n*=15; *RSK^Δ58/1^*=76±7, *n*=14, rank sum test *P*=0.527 versus wild type; *RSK^Δ58/1^;P[RSK]*=70±4, *n*=15, rank sum test *P*=0.213 versus wild type). Thus, basal properties of neurotransmitter release were not affected by loss of RSK. Instead, the electrophysiological results support a postsynaptic functional role for RSK at the glutamatergic *Drosophila* NMJ.

### RSK is required for axonal transport

**Fig. 7.**
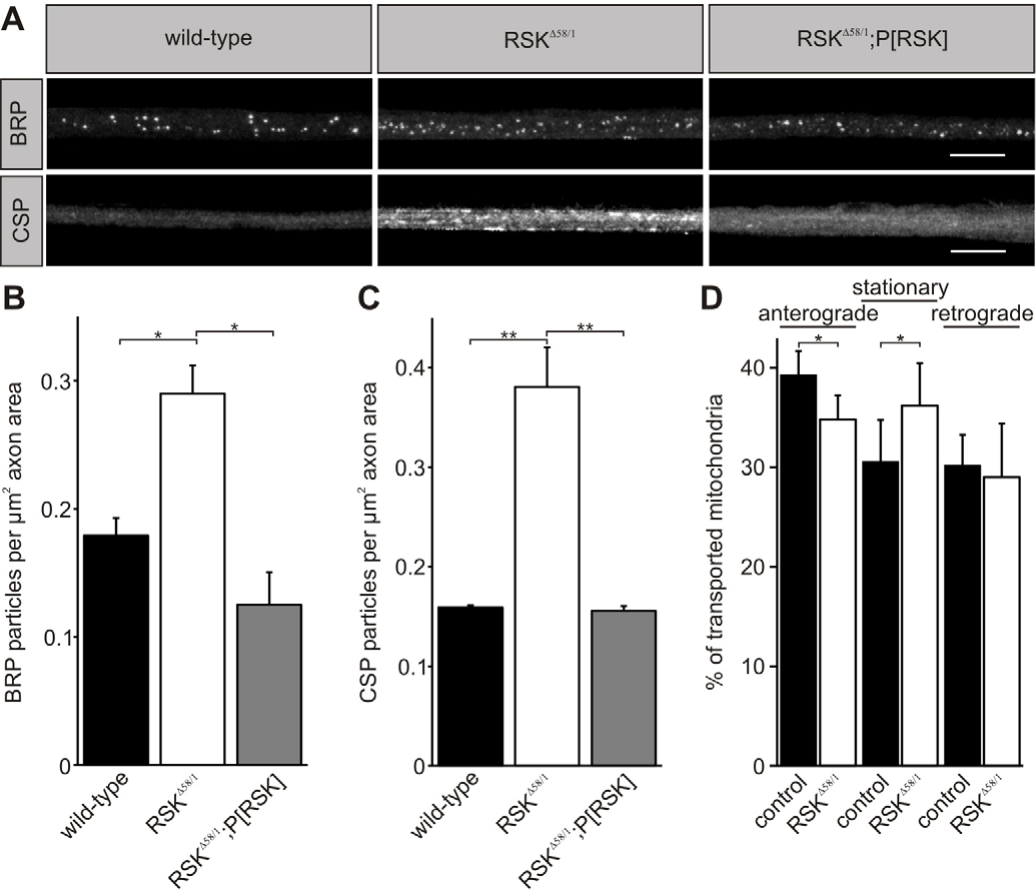
**Loss of RSK causes proximal accumulations of synaptic proteins and transport defects.** (A) Motoneuron axons from wild type, *RSK^Δ58/1^* and *RSK^Δ58/1^;P[RSK]* were stained for Bruchpilot (BRP; upper row) and cysteine string protein (CSP; lower row). Scale bar: 20 µm. (B,C) Quantifications for BRP (B; *n*=5 individual larvae per genotype) and CSP particles (C; *n*=8 individual larvae per genotype). (D) Analysis of the axonal transport of mitochondria in motoneurons by *in vivo* imaging. After bleaching of a length of axon, the movement of mitochondria was recorded in anesthetized larvae. Plots display anterograde- and retrograde-transported and stationary mitochondria in control animals (*OK6-Gal4;UAS-mito::GFP*, *n*=15) and the *RSK* mutant (*RSK^Δ58/1^;OK6-Gal4;UAS-mito::GFP*, *n*=10). *P≤0.05 and **P≤0.01.

The morphological analysis of *RSK* mutant motoneurons revealed another interesting phenotype. Compared with wild-type animals, an increased number of BRP particles were observed in segmental nerves of *RSK^Δ58/1^* larvae (Fig. 7A). This phenotype was most evident in proximal regions close to the ventral nerve cord but not in distal regions near synaptic termini and could be rescued by the *P[RSK]* transgene. Quantification of BRP puncta (Fig. 7B) revealed significant differences between *RSK^Δ58/1^* (0.290±0.022 BRP particles per µm^2^ axon area) and either wild-type (0.179±0.014 BRP particles per µm^2^ axon area; *P*<0.05) or *RSK^Δ58/1^;P[RSK]* animals (0.125±0.026 BRP particles per µm^2^ axon area; *P*<0.05).

Presynaptic development and function relies on coordinated assembly, transport and delivery of synaptic vesicle precursors, pre-assembled synaptic cytomatrix proteins and mitochondria (Goldstein et al., 2008; Maday et al., 2014; Maeder et al., 2014). To investigate whether loss of RSK specifically affects BRP or also affects other synaptic components, analyses with a synaptic vesicle-associated protein, cysteine string protein (CSP; Zinsmaier et al., 1994), were performed (Fig. 7A). CSP is associated with the cytoplasmic surface of synaptic vesicles and transported by synaptic vesicle precursors. Quantifications showed a significant increase of CSP accumulations in motoneuron axons of *RSK^Δ58/1^* larvae (Fig. 7C; 0.381±0.040 CSP particles per µm^2^ axon area) in comparison to wild-type animals (0.159±0.002 CSP particles per µm^2^ axon area; *P*<0.01) or *RSK^Δ58/1^* animals carrying the *P[RSK]* rescue construct (0.156±0.005 CSP particles per µm^2^ axon area; *P*<0.01).

The abnormal enrichment of BRP and CSP in proximal nerve regions could indicate impairment in assembly or transport of cargoes to the synaptic terminals. Microtubule-based transport in the anterograde direction by plus-end-directed Kinesin motors and in the retrograde direction by minus-end-directed Dynein motor proteins are essential for neuronal function and homeostasis. Disturbances of these processes have been implicated in the pathogenesis of several neurological disorders (De Vos et al., 2008; Hirokawa et al., 2010; Maday et al., 2014).

Given that we were unable to follow transport of a fluorescent-tagged variant of BRP in motoneurons reliably by *in vivo* time-lapse imaging (K.B., data not shown), we monitored bidirectional mitochondrial transport. Motility patterns of mitochondria are complex, with phases of fast movement, abrupt changes in direction of movement and stationary phases, and they rely on physiological changes (Zinsmaier et al., 2009; Sheng and Cai, 2012). Mitochondrial transport in motoneurons of living and intact wild-type or *RSK^Δ58/1^* third instar larvae was measured by expression of the mitochondrial GFP marker construct *UAS-mito::GFP* with the specific motoneuron driver *OK6-Gal4*. After photobleaching of an axon stretch, mitochondrial movements were recorded within a recovery period of 12 min and an imaging frequency of 720 ms. Kymographs were analyzed for anterograde, retrograde and stationary mitochondria (Fig. 7D). In axons of *RSK*-deficient larvae, fewer mitochondria were transported in the anterograde direction compared with control larvae (*OK6-Gal4;UAS-mito::GFP*=39.3±2.4%; *RSK^Δ58/1^;OK6-Gal4;UAS-mito::GFP*=34.8±2.5%; *P*<0.05), and the percentage of stationary mitochondria was significantly increased (*OK6-Gal4;UAS-mito::GFP*=30.6±4.2%; *RSK^Δ58/1^;OK6-Gal4;UAS-mito::GFP*=36.2±4.3%; *P*<0.05). These effects are specific to anterograde transport, because transport of mitochondria in retrograde direction was unaffected (*OK6-Gal4;UAS-mito::GFP*=30.2±3.1%; *RSK^Δ58/1^;OK6-Gal4;UAS-mito::GFP*=29.0±5.4%; *P*=not significant).

Taken together, loss of RSK function affects anterograde transport of mitochondria and leads to a biased accumulation of synaptic vesicle precursors and cytomatrix components in axon regions close to the ventral nerve cord.

## DISCUSSION

One emerging common picture from studies in animal models for CLS is that loss of RSK2 function in neurons is associated with deregulation of ERK signaling and synaptic properties. In general, ERK signaling is not only required for cell proliferation, differentiation and survival, but also for synaptic plasticity and memory (Thomas and Huganir, 2004; Davis and Laroche, 2006). The molecular functions of RSK proteins as an interaction partner of ERK proteins are discussed ambivalently in the literature. On the one hand, RSK2 mediates ERK signaling by phosphorylation of numerous targets; on the other hand, it is described as a negative regulator of ERK (Romeo et al., 2012). This ambivalent picture is also reflected by our genetic interaction experiments between *RSK* and *ERK* mutants, which did not provide a conclusive answer about the relationship between RSK and ERK with respect to pre- and postsynaptic functions. Further complexity is added because subcellular localization of pERK was changed in *RSK^Δ58/1^* motoneurons, with elevated pERK levels in the somata and strongly decreased levels at the NMJ. Thus, even in a single cell, opposing effects with respect to ERK targets in different subcellular compartments can be expected.

Our results coincide at several points with findings in the vertebrate nervous system. Elevated pERK levels were observed in the hippocampus of *RSK2* knockout mice, resulting in deregulation of ERK-mediated gene transcription (Mehmood et al., 2011; Schneider et al., 2011). For instance, transcription of the *Gria2* gene encoding the GLUR2 subunit of the AMPA receptor is upregulated. Nevertheless, electrophysiological, biochemical and ultrastructural analyses carried out with isolated cortical neurons and in the hippocampus revealed impaired AMPA-receptor-mediated synaptic transmission. This can be explained, at least in part, by the requirement of RSK2 for phosphorylation of postsynaptic PDZ [post synaptic density protein-95 (PSD95), discs large 1 (DLG1), zonula occludens-1 (ZO1)] domain-containing proteins to regulate channel properties (Thomas et al., 2005; Morice et al., 2013). Our morphological and electrophysiological data at the NMJ are also consistent with a postsynaptic requirement of RSK for synaptic transmission. In addition, *RSK* mutants displayed a number of defects in the presynaptic motoneuron, including upregulation and relocalization of pERK and a reduction in active zone numbers. How do these phenotypes relate to known functions of ERK in *Drosophila* motoneurons? First, alterations in ERK activity at the NMJ are inversely correlated with levels of the neural cell adhesion molecule Fasciclin II (Koh et al., 2002). Given that Fasciclin II was found to be excluded from pERK-positive spots at synapses, a direct regulatory mechanism at the protein level seems plausible. Thus, it is conceivable that synaptic RSK contributes to Fasciclin II-mediated cell adhesion either directly, by acting as an upstream kinase, or by feedback inhibition of ERK activity. Second, besides RSK, the serine-threonine kinase UNC-51 also acts as a negative regulator of ERK in motoneurons (Wairkar et al., 2009). It could therefore be expected that *RSK* and *UNC-51* mutations display similar synaptic phenotypes. Indeed, NMJ size, number of active zones and eEPSC amplitudes are decreased in both mutants. Interestingly, transgenic rescue experiments for the electrophysiological defects failed to work in both mutants, emphasizing the importance of fine-tuning ERK activity for maintaining normal synaptic functions. However, there are also significant differences between the two mutants. In general, *UNC-51* phenotypes are much more pronounced. In the *UNC-51* mutant, many postsynaptic GluRs are unapposed to presynaptic BRP, a phenotype we did not observe in the case of loss of RSK. Both mutants showed a decrease in eEPSC amplitude, but although this was attributable to defective transmitter release at *UNC-51* mutant synapses, no such presynaptic defect was observed in *RSK* mutants (unchanged quantal content; Fig. 6C). Instead, the reduced mini amplitude at *RSK* mutant synapses indicates impaired postsynaptic sensitivity, which in turn was unaltered in *UNC-51* mutants. Thus, although RSK and UNC-51 act as negative regulators for ERK, their relative contribution to ERK signaling in different cell types appears to be different. At least in the case of the *RSK* mutant, hyperactivation of ERK is modest and has no effect on development or viability of the fly, which implies a subtle modulatory function of RSK.

Finally, our analyses uncovered aberrant axonal BRP and CSP localization and anterograde transport defects of mitochondria. Transport of presynaptic components and their appropriate delivery at synaptic terminals require a complex interplay between motor proteins, the different transported components and local signaling events. In addition, mechanisms must exist to restrain localization of presynaptic components at the nerve terminals (Goldstein et al., 2008; Maeder et al., 2014). Interfering with these processes in *Drosophila* motoneurons caused distinct phenotypes. For instance, loss of Liprin-α results in ectopic accumulation of synaptic vesicles and presynaptic cytomatrix proteins in distal axon regions close to the synaptic terminals without affecting mitochondria or motor protein localization (Li et al., 2014). SR protein kinase 79D (SRPK79D) is required to prevent formation of large axonal agglomerates of BRP. Given that axonal transport processes and other synaptic proteins are not affected in *SRPK79D* mutants, a function of this kinase for site-specific active zone assembly at presynaptic membranes has been suggested (Johnson et al., 2009; Nieratschker et al., 2009). Large organelle-filled axonal swellings were observed in mutants defective for motor protein components; however, these aggregates do not serve as physical barriers for mitochondrial transport (Pilling et al., 2006). Local effects caused by changes in axonal transport are seen in *dAcsl* mutations. Here, mitochondrial transport is unaffected, but an increased velocity of anterograde transport and reduced velocity of retrograde transport of vesicles results in aggregates in distal axon regions (Liu et al., 2011). Mutation of the human ortholog *ACSL4* (acyl-CoA synthase long chain family member 4) causes non-syndromic X-linked mental retardation.

The axonal phenotypes seen in *RSK* mutants differ in several respects from these phenotypes. Large axonal swellings are not evident, and the increase in the number of BRP and CSP particles is largely confined to the proximal portion of the nerve (close to the ventral nerve cord). Together with the finding of more stationary mitochondria and fewer mitochondria transported in the anterograde direction, one explanation could be a function of RSK at the level of motor-cargo interaction. Specificity of cargo transport in the anterograde direction is determined at the levels both of individual Kinesins and of cargo-specific adaptor proteins (Hirokawa et al., 2010; Maday et al., 2014). For example, the catalytic subunit Kinesin-1 in *Drosophila* (kinesin heavy chain, KHC) recruits mitochondria via the adaptor protein Milton, whereas UNC-76 provides a link to the synaptic vesicle protein Synaptotagmin (Stowers et al., 2002; Gindhart et al., 2003). Motor-cargo interactions are also regulated in a phosphorylation-dependent manner, as exemplified by the UNC-51 kinase-dependent interaction of UNC-76 with Synaptotagmin (Toda et al., 2008). Loss of either UNC-51 or UNC-76 resulted in accumulations of synaptic vesicles along motoneuron axons (Toda et al., 2008). Another example is glycogen synthase kinase 3 (GSK-3), which has been proposed to inhibit anterograde transport by phosphorylating Kinesin light chain and thereby causing dissociation of membrane-bound organelles from KHC (Morfini et al., 2002). Based on genetic analyses in *Drosophila*, an alternative model proposes a function of GSK-3 in regulating motor protein activity rather than cargo binding (Weaver et al., 2013). Interestingly, RSK2 has been reported to inhibit GSK-3 activity in different cellular contexts and is able to phosphorylate GSK-3, at least *in vitro* (Romeo et al., 2012). Future studies are required to clarify a function of RSK in GSK-3-mediated control of anterograde transport processes. So far, we have no evidence for a direct or an indirect requirement of RSK for phosphorylation of motor protein components and, if so, whether this might have an impact on their *in vivo* function.

In summary, an emerging common picture from knockout studies in mice and flies as animal models for CLS is a postsynaptic requirement of RSK proteins for efficient synaptic transmission. In addition, we uncovered changes in the presynaptic neuron; in particular, defects in anterograde axonal transport and changes in localization of activated ERK. Whether these phenotypes reflect independent functions of RSK or whether they are interdependent remains to be determined. Future studies will also have to aim at understanding the function of RSK at central brain synapses in learning and memory processes.

## MATERIALS AND METHODS

### Fly strains and genetics

Flies were reared on standard cornmeal food at 25°C and 60% relative humidity in a 12 h-12 h dark-light cycle. Two viable *RSK* deletion mutants were used: *Df(1)ign^Δ58/1^* (in the following, referred to as *RSK^Δ58/1^*; Putz et al., 2004) and *RSK^D1^* (a kind gift from J. Chung, Seoul National University, South Korea; Kim et al., 2006). Other fly stocks used were as follows: *rl^1^*, a viable hypomorphic allele of *Drosophila* ERK (Biggs et al., 1994) and *rl^Sem^*, a dominant hyperactivated ERK variant (Brunner et al., 1994), *D42-Gal4* (Yeh et al., 1995), *DMef2-Gal4* (Ranganayakulu et al., 1998), *OK6-Gal4* (Aberle et al., 2002), *UAS-mCD8::GFP* (Lee et al., 1999) and *UAS-Mito::GFP* (Pilling et al., 2006); *w^1118^* was used as wild type. For generation of *UAS-GFP::RSK* transgenic flies, the open reading frame of *RSK* was amplified by linker PCR from cDNA clone SD05277 (Drosophila Genomics Resource Center) and cloned into the XbaI/NotI sites of a modified pUAST-vector (Brand and Perrimon, 1993) 3′ to the coding sequence of eGFP. Transgenic lines were established by BestGene Inc. (Chino Hills, CA, USA). For rescue experiments, clone CH322-75N12, which contains a 20 kb genomic fragment encompassing the *RSK* gene locus, was chosen from a genomic BAC library engineered into the attB-P[acman]-Cm^R^-BW vector (Venken et al., 2009). Transgenic flies for this clone (in the following, named *P[RSK]*) were generated by PhiC31-mediated integration at the *attP* site located at chromosomal position 65B2 on the third chromosome (BestGene Inc.).

### Western blot

For quantification of ERK and pERK levels, 50 ventral ganglia from late third instar larvae were dissected in ice-cold fixation solution (4% paraformaldehyde in PBS) to minimize changes in phosphorylation status during handling, fixed for 10 min on ice and washed three times for 20 min in PBS. Following homogenization and sonication, protein lysates were analyzed by western blotting with the following antibodies: monoclonal rabbit anti-Phospho-p44/42 (ERK1/2, Thr202/Tyr204; clone D13.14.4E, 1:2000; Cell Signaling), monoclonal rabbit anti-p44/42 (ERK1/2; clone 37F5, 1:1000; Cell Signaling) and mouse anti-α-tubulin (clone DM1a, 1:10,000; Sigma-Aldrich). Sheep anti-mouse-HRP (1:10,000; GE Healthcare) and donkey anti-rabbit-HRP (1:10,000; GE Healthcare) were used as secondary antibodies. Western blots were scanned with an Intas ChemoCam imager, and intensities of protein bands were measured with the ImageJ 1.48a software (NIH, Bethesda, MD, USA). The intensity of the α-tubulin band was used for normalization.

### Immunohistochemistry

Wandering third instar larvae were dissected in HL-3 medium as described previously (Brent et al., 2009) or directly in fixation solution in the case of anti phospho-ERK staining to minimize dephosphorylation. Depending on the experiment, combined brain and body-wall preparations or only body walls were dissected. Fixation was done for 15 min in 4% (w/v) paraformaldehyde at room temperature. After washing in PBT [PBS+0.05% (v/v) Triton X-100] and blocking in PBT supplemented with 3% (v/v) normal goat serum for 30 min, samples were incubated overnight at 4°C with combinations of the following antibodies diluted in PBT supplemented with 3% normal goat serum: chicken anti-GFP (1:1000; Merck Millipore), rabbit anti-Glutamate receptor subunit GluRIID (Qin et al., 2005; 1:1000; a kind gift from S. Sigrist, University of Berlin, Germany), mouse anti-Bruchpilot (Wagh et al., 2006; anti-BRP^nc82^, 1:100; a kind gift from E. Buchner, University of Würzburg, Germany), mouse anti-Cysteine string protein (Zinsmaier et al., 1994; anti-CSP^ab49^, 1:100; E. Buchner), mouse anti-lamin Dm0 (clones ADL67.10 and ADL195, both at 1:10; Developmental Studies Hybridoma Bank), rabbit anti-Phospho-p44/42 (ERK1/2, Thr202/Tyr204; clone D13.14.4E, 1:200; Cell Signaling) and goat anti-HRP Cy5-conjugated (1:250; Dianova). Secondary antibodies were conjugated with AlexaFluor 488, DyLight488, Cy3 or Cy5 (Dianova) and used at dilutions of 1:100 to 1:200 in PBT. After washing in PBT, preparations were embedded in VectaShield (Vector Laboratories).

### Imaging and analysis of fixed samples

Confocal images were recorded using either an Olympus Fluoview 1000 IX 81 or a Leica SP5 microscope. Images were processed using ImageJ 1.48a (NIH, Bethesda, MD, USA). To avoid variations within a single experiment, all preparations of larvae from different genotypes were stained simultaneously as described by Viquez et al. (2006). All genotypes in one experiment were imaged with the same gain, avoiding saturation of the signal. For imaging and quantification of NMJ size, areas and numbers of active zones and postsynaptic densities at muscle 6/7 in abdominal segment A2 or A3, we exactly followed the step-by-step protocol of Andlauer and Sigrist (2012c) with the following minor modification: threshold values for detection of active zones and GluR fields were set to equal values in all experiments to allow for semi-automated quantification and comparison of different genotypes. For quantification of BRP and CSP aggregates, images were taken from axons that emerge from the ventral ganglion at segment A3, using the same gain for all genotypes. Numbers of particles for BRP or CSP were normalized to the axon area outlined by HRP staining.

### *In vivo* imaging

Examination of axonal transport in living larvae was carried out in an imaging chamber as described by Andlauer and Sigrist (2012a,b) using an Olympus Fluoview 1000 IX 81 confocal microscope equipped with a 60× oil, NA 1.35 objective. After photo-bleaching of an axon length of 61.44 µm, ∼100 µm distal to the tip of the ventral ganglion, series of 1000 frames were scanned with imaging intervals of 720 ms. The series were processed in ImageJ 1.48a and analyzed based on kymographs generated in MATLAB (R2010b).

### Data analysis

Statistical analyses of electrophysiological results were performed with the non-parametric rank sum test (Sigma Plot 12.5; Systat Software). For all other data, Mann-Whitney *U*-tests were used for statistical analyses. For multiple testing within one data set, the level of significance *P*<0.05 was adjusted according to the Bonferroni correction. Data are reported ±s.e.m., and asterisks depict level of statistical significance as follows: *P≤0.05, **P≤0.01 and ***P≤0.001.

### Electrophysiology

Two-electrode voltage-clamp recordings (Axoclamp 900A amplifier; Molecular Devices) were made from muscle 6, segments A2 and A3 of late third instar male *Drosophila* larvae essentially as previously reported (Ljaschenko et al., 2013). All measurements were performed at room temperature in extracellular haemolymph-like solution (HL-3; Stewart et al., 1994) containing (in mM): NaCl, 70; KCl, 5; MgCl_2_, 20; NHCO_3_, 10; trehalose, 5; sucrose, 115; HEPES, 5; CaCl_2_, 1; pH adjusted to 7.2. Intracellular electrodes with resistances of 10-20 MΩ (filled with 3 M KCl) were used, and only cells with an initial membrane potential of at least −50 mV and a membrane resistance ≥4 MΩ were taken into consideration. During recordings, cells were clamped at a holding potential of −80 mV for minis and −60 mV for eEPSCs. To evoke synaptic currents, nerve stimulation (300 μs pulses, typically at 10 V; Grass S88 stimulator and isolation unit SIU5; Astro-Med) was applied via a suction electrode (diameter ∼15 µm; filled with extracellular solution). Signals were sampled at 10 kHz, low-pass filtered at 1 kHz and analyzed using Clampfit 10.2 (Molecular Devices). Quantal content was calculated by dividing the average eEPSC amplitude by the average mini amplitude, corrected for the more hyperpolarized holding potential (amplitude reduction to 75%; Hallermann et al., 2010).
